# Complications of Bezoar in Children: What Is New?

**DOI:** 10.1155/2013/523569

**Published:** 2013-10-29

**Authors:** Kam Lun Ellis Hon, Jean Cheng, Chung Mo Chow, Hon Ming Cheung, Kam Lau Cheung, Yuk Him Tam, Alexander K. C. Leung

**Affiliations:** ^1^Prince of Wales Hospital, Shatin, Hong Kong; ^2^Department of Paediatrics, The Chinese University of Hong Kong, Prince of Wales Hospital, Shatin, Hong Kong; ^3^The University of Calgary, Alberta Children's Hospital, Calgary, Alberta, Canada

## Abstract

A bezoar is a mass found trapped in the gastrointestinal system. The condition may be associated with pica, especially in developmentally retarded children. Clinical manifestations are usually nonspecific. Endoscopic diagnosis and removal of the foreign materials is often indicated. Occasionally, severe complications may occur. We report two cases to illustrate the clinical features and complications in these children. In the first case, a reliable history was not obtained in the developmentally delayed girl which precluded prompt diagnosis, but the grossly dilated stomach on plain abdominal radiograph gave clues to an underlying insidious mechanical obstruction of upper gastrointestinal tract. In the second case of a normal child, the unrelenting symptoms and weight loss prompt further investigations which revealed the diagnosis. Literature on pediatric bezoar is reviewed. Oesophagoduodenoscopy is the investigation of choice for diagnostic confirmation, but surgical facilities must be available to deal with acute complications.

## 1. Introduction

A bezoar (from Persian bazahr, “antidote”) is a mass found trapped in the gastrointestinal system (usually the stomach), though it can occur in other locations [1–4]. A bezoar in the esophagus or upper gastrointestinal tract is commonly reported in developmentally delayed or institutionalized children. We report two cases to illustrate different salient features and management challenges of this disease. Bezoars are usually associated with symptoms from local obstruction or suffocation. A high index of suspicion with prompt diagnosis and treatment is pivotal to minimize complications. Occasionally, complications may develop during the surgical procedure to remove the impacted materials.

## 2. Case 1

**Figure 1:**
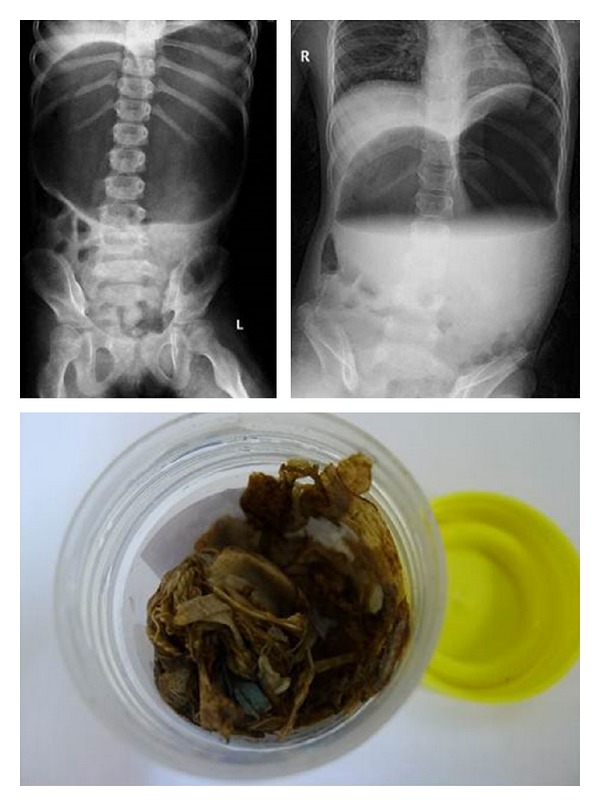
Grossly distended stomach by radiography and debris (gauze and adhesive tape) removed in this child with 46XX del (11).

A developmentally delayed girl from Mainland China was abandoned at the age of 3 years in the streets of Hong Kong and subsequently institutionalized. She had multiple abnormalities including surgery for presumed Hirsprung disease in Mainland China. She was confirmed to have 46XX del (11) chromosomal abnormality in Hong Kong. She resided at an institution, and reportedly, she had pica and chronic constipation. At age of 9 years, she was admitted with painless abdominal distension. Abdominal radiography showed dilated gastrium (Figure 1). The patient was then referred to a teaching hospital for elective oesophagogastroduodenoscopy which revealed large amounts of debris and gauze impacted at the stomach antrum. Approximately, 60% of the debris was removed with 20 passages of grasper. The procedure was complicated by gastric perforation at posterior wall of fundus, with resultant pneumoperitoneum and difficulty ventilation, which was emergently drained by a 14-gauge catheter. Emergency operation was carried out. Gastric perforation of posterior wall from fundus to greater curvature with necrotic edge and multiple serosal tears on both sides of perforation were repaired. Postoperatively, she was transferred to the Pediatric Intensive Care Unit (PICU) for further care. She required ventilatory supports, intravenous antibiotics, transfusions of packed cells and fresh frozen plasma for anemia (lowest hemoglobin 9.1 g/dL), and coagulopathy (prothrombin time 22.5 seconds and activated partial thromboplastin time 41.3 seconds). The patient was monitored in PICU for 3 days before discharge to the paediatric surgery. Peritoneal swab yielded heavy growth of penicillin-sensitive *streptococcus pneumoniae*.

## 3. Case 2

**Figure 2:**
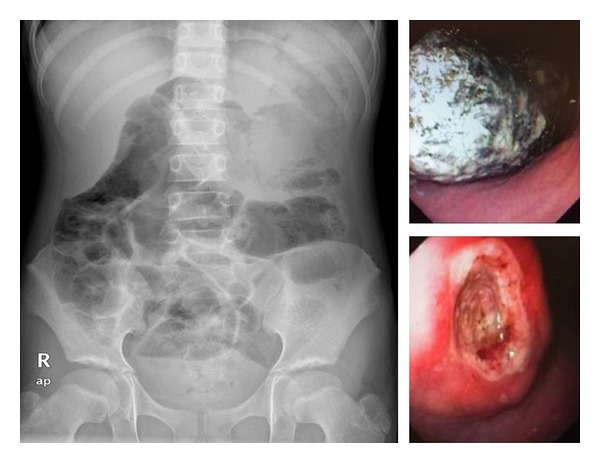
Dilated stomach and small bowel loops. A large trichobezoar and a gastric ulcer were visualized by oesophagogastroduodenoscopy.

A 9-year-old girl with good past health presented with unrelenting left-sided abdominal pain, nausea, and vomiting undigested foods (4-5 times per day) for 3 weeks. There were no symptoms of acid reflux, altered bowel motility, blood, or mucus in stool. Her body weight dropped from 27.3 kg to 25.8 kg (10–25% centile) over 6 weeks as a result of poor oral intake and vomiting. Left upper quadrant and epigastric fullness were noted. Blood tests showed iron deficiency anaemia with hemoglobin of 9.2 g/dL. Abdominal radiography showed nonspecific dilatation of gastrum and small bowel loop (Figure 2). Oesophagogastroduodenoscopy revealed that a large foreign body occupied nearly the whole stomach and two chronic ulcers measuring 2-3 cm at the angular and lesser curve (Figure 2). Biopsies were taken which later confirmed chronic inflammation, focal erosion, and ulcer but no evidence of *helicobacter pylori* infection. Subsequently, a 16 cm long trichobezoar was removed piecemeal via gasrtrostomy following laparoscopy and minilaparotomy. The clinical course was complicated by abdominal wound dehiscence which necessitated delayed secondary suturing of wound one month later. Child psychiatry assessment revealed no evidence of psychiatric illness or social problems.

## 4. Discussion

Many cases of bezoars have been reported in children who are mentally retarded or having psychosocial problems [4–10]. Nevertheless, the condition can occur in normal children with no apparent psychosocial issues [5, 8]. In the first case, a reliable history was not obtained in the developmentally delayed girl with 46XX del (11) chromosomal anomaly which precluded prompt diagnosis, but the grossly dilated stomach on plain abdominal radiograph gave clues to an underlying insidious mechanical obstruction of the upper gastrointestinal tract. In the second case, the unrelenting symptoms and weight loss prompted further investigations which revealed the diagnosis. The girl had normal intelligence and no associated psychiatric illness or social problems for her bizarre behavior.

A literature search using the keyword “bezoar” and with limits activated (humans and ages from birth to 18 years) was performed using PubMed in August 2013. Journal impact factor (IF) is in accordance with Journal Citation Report (JCR), a product of Thomson (ISI) Institute for Scientific Information. 635 publications were retrieved. The majority of these publications were single case reports with relatively low impact factors. With additional filter activated (reviews), 48 publications were obtained, most are case reports with review of the subject matter. With filter “clinical trials” activated, 3 publications were retrieved, but only one was relevant [11]. The authors report on their experience in fragmenting huge, solid bezoars using a modified needle-knife (bezotome) and a modified mechanical lithotriptor (bezotriptor) on 15 patients (ten male, five female, median age 41 years) with 17 gastric bezoars and one esophageal bezoar, treated endoscopically. All 18 bezoars were successfully fragmented, ten in one session and eight in two sessions. Complete clearance of the upper digestive tract was achieved at the latest three days after the treatment. There were no complications. The authors conclude that bezotome and bezotriptor are useful endoscopic devices to disintegrate huge, hard bezoars and achieve complete clearance.

Bezoar is an easily missed diagnosis especially in the mentally retarded young patients [1, 10]. Symptoms of bezoar can mimic many gastrointestinal diseases [4]. Localized signs such as epigastric fullness may be subtle [4]. Hence, high index of suspicion and further investigations are invariably required to confirm the diagnosis. Moreover, growth parameters should be measured to evaluate any malnutrition or stunted growth. Radiologic investigations include abdominal radiography to reveal any distended gastric antrum, with associated dilated small bowel loop (Rapunzel Syndrome), and chest radiography to reveal any air under the diaphragm signifying intestinal perforation [1, 5]. Ultrasound abdomen can be performed to evaluate the nature, size, and position of the mass [4]. Computerized tomography could also be performed to better delineate the mass [12]. 

Oesophagogastroduodenoscopy is indicated for definitive diagnostic and therapeutic purposes [6–10]. The procedure enables direct visual assessment of the mass and any ulcers. Therapeutically, it could remove the mass and treat the associated ulcers. However, oesophagogastroduodenoscopy is not always effective and at time associated with risk of intestinal perforation. Laparoscopy or laparotomy may be required [4, 8]. Laparotomy as described by Gorter et al. carried a 100% successful rate [8]. Nevertheless, wound complications or dehiscence may result. 

Complications of bezoar and its treatment include weight loss, malnutrition, anemia, gastric ulcer, bowel obstruction, and surgical complications [1, 4, 9, 10, 13]. Gastric perforation, coagulopathy, and pneumococcal peritonitis are reported in the first case, while weight loss, malnutrition, ulcers, and wound dehiscence are described in the second. 

In summary, bezoar should be considered as a diagnostic differential when a developmentally delayed child with history of pica presents with acute abdominal symptoms. The symptoms might be confused with gastroenteritis. A high index of suspicion with prompt diagnosis is pivotal to avoid unnecessary nonspecific treatment and delay in surgery for this disease. Oesophagogastroduodenoscopy allows definitive diagnosis to be made in a timely manner.
